# Features of chinese patients with sitosterolemia

**DOI:** 10.1186/s12944-021-01619-1

**Published:** 2022-01-18

**Authors:** Zhizi Zhou, Xueying Su, Yanna Cai, Tzer Hwu Ting, Wen Zhang, Yunting Lin, Aijing Xu, Xiaojian Mao, Chunhua Zeng, Li Liu, Xiuzhen Li

**Affiliations:** 1 Department of Genetics and Endocrinology, Guangzhou Women and Children’s Medical Center, 9 Jinsui Road, 510623 Guangzhou, Guangdong China; 2 Department of Pediatrics, Faculty of Medicine & Health Sciences, University Putra Malaysia, 43400 Serdang, Selangor, Malaysia

**Keywords:** Sitosterolemia, Xanthoma, Hypercholesterolemia, *ABCG5*, *ABCG8*

## Abstract

**Background:**

Sitosterolemia is a lipid disorder characterized by the accumulation of phytosterols in plasma and organs, caused by mutations in the *ABCG5* and/or *ABCG8* genes. The disease is frequently misdiagnosed and mistreated as familial hypercholesterolemia (FH). To gain a better understanding of the disease, the current status of diagnosis and treatment of Chinese patients with sitosterolemia was reviewed and summarized.

**Method:**

Literature search was performed. The clinical features and molecular characteristics of Chinese patients with sitosterolemia were analysed. Four children with sitosterolemia and the treatment experience were described.

**Results:**

Fifty-five patients with sitosterolemia have been reported in China. These patients were aged from 3 months to 67 years at diagnosis, and the median was 8 years of age. Several complications, such as xanthomas in 47 patients (85%), thrombocytopenia in 17 patients (31%), anemia in 14 patients (25%), and cardiovascular damage in 12 patients (22%), were observed. Thirty-nine patients (71%) exhibited mutations in the *ABCG5* gene, 15 patients (27%) showed mutations in *ABCG8*, and variations in both genes occurred in one patient (2%). A patient with two clinically rare diseases, namely, sitosterolemia and glycogen storage disease type VI (GSD VI)), is reported here for the first time. The four reported patients were treated with low cholesterol and phytosterol-limited diet alone or combined with cholestyramine. Even though decreases were observed for total plasma cholesterol (TC) and low-density-lipoprotein cholesterol (LDL-C), and these levels were as low as normal in some patients, the levels of plant sterols remained above the normal range. However, TC, LDL-C and plant sterol levels remained at high levels in patients treated with a control diet control only.

**Conclusions:**

The analysis reveals that different from Caucasians carrying mainly variations in ABCG8, most Chinese patients have mutations in the *ABCG5* gene, and Arg446Ter, Gln251Ter, anArg389His might be hot-spot mutations in Chinese patients. The current survey provides clinical data to enable the development of a standardized protocol for the diagnosis and treatment of sitosterolemia in China.

## Background

Sitosterolemia (MIM# 210,250) is a rare autosomal recessive hereditary metabolic disease and a lipid metabolism disorder characterized by increased absorption and decreased excretion of dietary phytosterols, primarily due to mutations in the *ABCG5* and/or *ABCG8* genes [1–3]. Sitosterolemia usually results in significantly increased plasma levels of phytosterols, namely sitosterol, cholestanol, campesterol and stigmasterol. Patients with sitosterolemia suffer from xanthomas, hypercholesterolemia, accelerated atherosclerosis, and liver dysfunction. Hematological abnormalities, including macrothrombocytopenia, hemolysis, anemia and stomatocytosis, have been reported [4, 5]. Sitosterolemia is highly heterogeneous clinically, varying widely from asymptomatic to premature cardiac death [6]. It is often misdiagnosed as familial hypercholesterolemia in the early stage, resulting in inappropriate dietary intervention consisting of plant-based foods high in phytosterol, leading to serious vascular complications [7]. Ezetimibe, a selective cholesterol absorption inhibitor, is currently used as a first-line agent to treat sitosterolemia patients in Western countries [8–10]. Although ezetimibe and cholestyramine have been widely used in the treatment of sitosterolemia, the unified consensus on the drug dosage and the intake of plant sterols during dietary therapy have not been reached, especially for infantile patients [11]. In China, the diagnosis and treatment of pediatric patients with sitosterolemia remain a challenge for clinicians. To expand the knowledge and reported information regarding clinical experiences related to sitosterolemia, the natural history and follow-up assays for four sitosterolemia children were traced and reported here. Literature review on clinical presentation and molecular characteristics of a total of 55 Chinese patients was conducted by collecting information from accessible databases, as well as internet sources, such as PubMed, Wiper database, Baidu Scholar, China National Knowledge Infrastructure (CNKI), and Wanfang Data [12–21].

Glycogen storage disease type VI (GSD VI) (MIM #232,700) is an autosomal recessive disease confirmed to be caused by mutations in the gene that encodes glycogen phosphorylase (*PYGL*), resulting in significantly elevated glycogen accumulations in the liver. The *PYGL* gene, located on chromosome 14 (14q22.1), consists of 20 exons. According to the Human Gene Mutation Database, 69 mutations have been reported in the *PYGL* gene. The clinical manifestations of this disease include hepatomegaly, growth retardation, mild hypoglycemia, hypertriglyceridemia, increased liver transaminases and ketosis in the early childhood stage. Muscular hypotonia and lactic acid elevation after meals have also been reported in some cases [22, 23]. Small frequent meals are recommended in all patients with GSD VI. Uncooked cornstarch has been proven to be effective in maintaining blood sugar stability and preventing ketosis, so patients are also recommended to eat uncooked cornstarch before bed [24].

## Case presentation

The four patients were from four separate healthy nonconsanguineous Chinese parents. All the patients had a normal birth history, except for case 4, who was conceived by *in vitro* fertilization (IVF). None had a family history of hypercholesterolemia, xanthoma, or early onset of cardiovascular diseases. Table 1 shows the clinical characteristics. All had normal complete blood cell counts and normal blood cell morphology on blood smears. Color Doppler ultrasonic studies revealed normal bilateral carotid artery blood flows in all patients. No abnormality was observed on electrocardiogram and echocardiography. The diagnosis of sitosterolemia was delayed in all four patients, up to six years and eight years, in patients 1 and 3 respectively.

**Table 1:** Baseline lipid profiles, liver enzymes, and blood cell counts of 4 Chinese children with sitosterolemia

	Normal values	Patient no			
		1	2	3	4
Gender		F	F	F	M
Mutation^a^ (*ABCG5*)		c.904+1G>A (p. Met302Asn fs*82) c.1568 C>A (p.His510Asn)	c.130T>C (p.Ser44Pro) c.1166G>A (p.Arg389His)	c.64 C>T (p.Gln22Ter) c.1568 C>A (p.His510Asn)	c.751 C>T (p.Gln251Ter) c.1166G>A (p.Arg389His)
Xanthomas		+	-	-	+
**Initial diagnostic data**					
Age at onset		3y	3y5m	3 m	1y5m
Age at diagnosis		9 y	4y8m	8y	2y
Serum Sitosterol, µmol/L	1~15	506	156	187	120
Campesterol, µmol/L	0.01~10	110	41	130	58
Liver enzymes					
Alanine transaminase, U/L	3~35	13	473	16	17
Asparate aminotransferase, U/L	5~60	29	349	40	38
Cholesterol, mg/dL					
Total	112~221	543	183	398	329
Low-density-lipoprotein	47~131	395	146	315	296
Total triglycerides, mg/dL	40~160	121	194	92	122
Blood count					
Erythrocytes, count/µL	(3.8~5.9)×10^∧^9	4.4	3.7	4.0	4.8
Hemoglobin, g/dL	110~151	129	108	111	109
Mean corpuscular volume, fl.	80~97	83.5	90.3	83.3	71
White blood cells, count/ul	5~12	5.2	3.1	4.9	10.7
Platelets, count/mm^3^	(140~440)×10^∧^6	230	211	279	270
**After treatment**					
Age at plant sterols analysis		11y	7y8m	10y10m	2y8m
Therapy		Cholestyramine +Diet	Diet	Cholestyramine +Diet	Diet
Cholesterol, total mg/dL	112~221	190	200	181	398
Low-density-lipoprotein	47~131	96	128	100	340
Serum Sitosterol, µmol/L	1~15	236	117	61	120
Campesterol, µmol/L	0.01~10	59	177	73	58

*Abbreviations: NA* not available, y year, m month

+ present, - absent

^a^ case2 combined with *PYGL* gene mutation

Case 1, a 12-year-old girl, presented with multiple xanthomas at her buttocks, wrists and ankles since she was three years old. The girl also exhibited a dramatically high level of total plasma cholesterol (TC) (624 mg/dL) and normal level of triglycerides (TGs). She underwent surgical excision of the xanthomas when she was 5 years old, and the histopathology examination confirmed xanthomas. She was initially misdiagnosed as FH and was treated with atorvastatin (5 mg/d) and diet modification (diet low in fat and cholesterol, high in fiber). The TC and low-density-lipoprotein cholesterol (LDL-C) levels remained elevated, and the xanthomas continued to increase in size and number.

Hence, she was referred for further evaluation at 9 years old. Height and weight were approximately in the last 25th percentile, and the body mass index was 14.9 kg/m^2^. Blood tests revealed normal levels of liver enzymes, blood glucose, coagulation parameters, and creatinine. Abdominal ultrasound showed no hepatosplenomegaly or fatty liver. Levels of serum plant sterols, such as β-sitosterol (506 µmol/L) and campesterol (110 µmol/L) were dramatically increased. Genetic analysis showed hybrid *ABCG5* gene mutant p.Met302Asn fs*82 (c.904+1G>A) and p.His510Asn (c.1568 C>A).

Treatment was changed to cholestyramine (1 g four times daily) combined with diet control (low in cholesterol and phytosterols). Xanthomas increased in the first 4 months, then decreased gradually, and finally almost disappeared after 1 year of treatment. The TC and LDL-C levels normalized after 1 year of treatment. Despite a 2-year cholestyramine treatment, her plasma plant sterol level remained high (Table 1).

Patient 2, showed elevated liver enzymes and enlarged liver size since 3 years old. When she was admitted to our center at 4 years and 8months old, her body height and weight (97.2 cm and 15.3 kg) were between the 3rd and 10th percentiles. The body mass index 16.2 kg/m^2^ was normal. Blood tests showed mild normochromic anemia with normal platelet count, markedly elevated levels of ALT and AST; moderate increase in lactic acid [1.81 mmol/L, normal range (NR) < 1.7 mmol/L] and *β*-hydroxybutyrate (2.69 mmol/L, NR: 0.02-1.8 mmol/L) levels. Blood glucose, coagulation parameters, and creatinine were normal. Serum lipid profiles showed increased LDL-C level (146 mg/dL), whereas the TG (194 mg/dL) and TC levels were normal. Serum albumin level (36.9 g/L) was mildly reduced (NR: 40-55 g/L). Color Doppler ultrasonography revealed hepatosplenomegaly with an oblique diameter of 97 mm in the right liver lobe. Liver biopsy demonstrated extensive water degeneration in the liver cells; however, no significant inflammatory cell infiltration or fibrosis was observed.

Genetic analysis by whole-exome sequencing (WES) detected compound heterozygous mutations in both the *PYGL* and *ABCG5* genes. Measurement of serum sterols by gas chromatography detected elevated β-sitosterol and campesterol levels (Table 1).

After the diagnosis was revised to GSD VI and sitosterolemia, oral cornstarch (1 g/kg per-dose, twice a day) was started, and the intake of cholesterol and plant sterols (<250 mg/day) was restricted. After two and a half years of treatment, the liver size, liver transaminase and TG levels normalized, and no signs of liver fibrosis were observed. However, there was no improvement in the blood sitosterol levels (Table 1). Cutaneous xanthomas were not observed within the entire treatment course.

Patient 3 had increased cholesterol levels since 7 years of age. On physical examination, both height and weight were on the 3rd percentile, and the body mass index (13.7 kg/m^2)^ was normal. Xanthoma was not found on the skin. Hypercholesterolemia was detected. Quantitative analysis of serum sterol levels showed very high *β*-sitosterol and campesterol concentrations (Table 1), indicating the occurrence of sitosterolemia. She was treated with cholestyramine, combined with a low-phytosterol diet. The serum cholesterol concentration normalized after four months of treatment. However, her serum sitosterol level remained high despite treatment for 2 years and 9 months (Table 1).

Patient 4, a 2-year-old boy, had cutaneous xanthomas on both ankles noted since 1 year and 5 months old. The preliminary laboratory examination indicated markedly elevated TC, LDL-C, and TG levels of 911 mg/dL, 762 mg/dL and 92 mg/dL, respectively. He was treated with dietary modification limiting milk (breast & formula) intake to 300-400 mL per day. Solid foods, mainly rice, meats, vegetables and one egg per day, were administered.

Upon referral to the tertiary center, his height and weight were in between the 10th and 25th percentiles, and the body mass index 15.2 kg/m^2^ was normal. He had multiple xanthomas at the elbows, ankles and gluteal folds. After the diagnosis of sitosterolemia was made based on elevated serum plant sterol levels, he received a low-cholesterol and low-phytosterol diet (avoiding vegetable oils, nuts, egg yolks and cereals, reducing milk to 150 mL per day, and excluding formula milk). Unfortunately, his serum cholesterol and sitosterol concentrations remained high after 8 months of dietary intervention (Table 1).

## Methods

The serum sitosterol levels were determined by a gas chromatography-mass spectrometric analyzer (GCMSQP2010 Plus, SHIMADZU, Japan), as described by Bratinčević MV et al. [25].

Genomic DNA (gDNA) was extracted from the peripheral blood of the patients as well as their parents using a DNeasy Blood & Tissue Kit ((Qiagen, Hilden, Germany). All the probands’ DNA samples were first amplified by PCR using specific primers for the ABCG5 and ABCG8 genes. The primers used for gDNA amplification and sequencing of ABCG5 and ABCG8 were designed by strictly following the descriptions in a previous article [26]. For WES (performed for patient 2) the manufacturer’s protocol was strictly followed for the entire workflow. The SureSelect Human All Exon V6 Kit (Agilent, California, USA) was used for library construction and capture experiments. DNA fragments were sequenced on the Illumina HiSeq 2500 platform. The captured variants were annotated by retrieval from SNP databases and the Human Gene Mutation Database (HGMD) (www.hgmd.cf.ac.uk). For novel variants absent from the HGMD, other existing databases, such as PROVEAN, SIFT, Mutation Taster, PolyPhen-2, Mutation Accessor and FATHMM, were used to predict pathogenicity.

Informed consent was obtained from all patients’ parents. The study was approved by the ethics committee of Guangzhou Women and Children’s Medical Center.

### Results of mutation analysis

No mutations were found in the ABCG8 gene. In the *ABCG5* gene, six mutations were identified, including 1 splicing mutation, 3 missense mutations and 2 nonsense mutations (Table 1). Among them, the splicing mutation c.904+1G>A (p.Met302Asn fs*82) located at exon 8 has been reported previously [27]. The mutation c.1568 C>A (p.His510Asn) located at exon 11 resulted in the replacement of histidine (His) by asparagine (Asn) at position 510. The mutation p.Ser44Pro (c.130T>C) located at exon 1 led to a change in serine (Ser) to proline (Pro) at position 44. The mutation p.Arg389His (c.1166G>A) located at exon 9 caused the replacement of arginine (Arg) with histidine (His) at position 389. Two nonsense mutations, c.64 C>T (p.Glu22Ter) and c.751 C>T (p.Gln251Ter), located at exon 1 and exon 6, respectively, initiated a stop codon of glutamine (Gln) at positions 22 and 251.

In addition to mutations in the *ABCG5* gene, we also found hybrid heterozygous mutations in the *PYGL* gene in patient 2. The mutation c.1149 A>C (p.Glu383Asp) located at exon 10 caused the replacement of the glutamic acid (Glu) residue at position 383 with aspartic acid (Asp). Another mutation was a gross deletion mutation of *PYGL* exons 14–17.

### Review of the Chinese patients

The relevant literature was searched in PubMed and Chinese databases, such as CNKI and Wanfang Data. According to the literature search, from year 2002 to 2020, 55 patients with sitosterolemia, have been reported in Taiwan and mainland China. These patients included 29 females and 26 males aged 3 months to 67 years, and the median age at diagnosis was 8 years. The *ABCG5* and/or *ABCG8* genes were analyzed for these 55 patients to confirm the diagnosis of sitosterolemia, serum sitosterol assays were performed for 40 of the 55 patients.

**Fig. 1:**
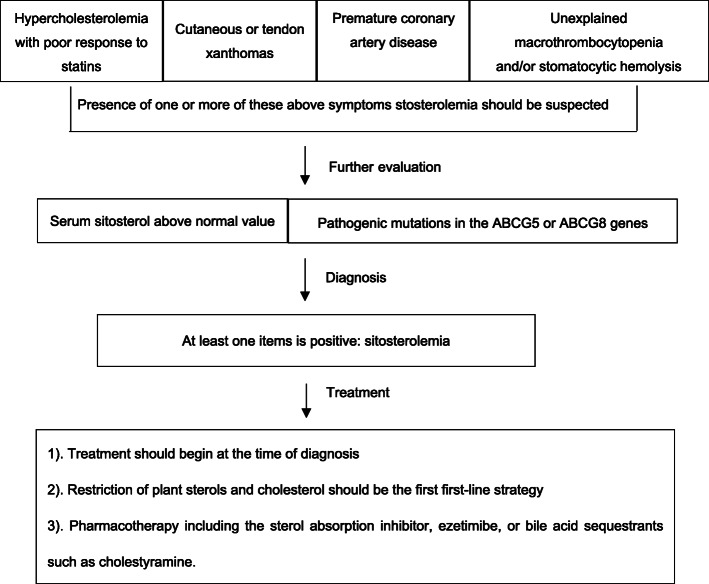
The proportion of clinical manifestations in 55 Chinese patients with sitosterolemia

Among these patients, the minimum age for symptom onset was 3 months (cases 3 and 4, a pair of siblings), and the maximum age at diagnosis was 67 years (patient 20). Xanthomas occurred in 47 patients (85%), thrombocytopenia in 17 patients (31%), anemia in 14 patients (25%), and cardiovascular damage (Table 2) in 12 patients (22%) (Fig. 1).

**Table 2:** A summary of clinical findings, laboratory serum lipid profiles, and *ABCG5*/*ABCG8* mutations in 55 Chinese sitosterolemia patients

Age at diagnoses (year)	Sex	Clinical Features				lipid profile			Gene mutation		Case (Ref)
		Xanthomas	Thrombocy-topenia	anemia	Cardiovascular damage	TC(mg/dL)	LDL-C (mg/dL)	SistosteroL (µmol/L)	*ABCG5*	*ABCG8*	
7	M	+^a^	-	-	-	709	636	2300.0	Arg419His/IVS12+1G>A		1(12)
8	F	+	-	-	-	427	346	NA	Tyr329Ter/Asn437Lys		2(13)
1.5	F	+	-	-	-	705	565	170.4	Arg389His/Arg446Ter		3(13)
0.25	F	-	-	-	-	402	304	220.1	Arg389His/Arg446Ter		Sibling of case3(13)
1.9	F	+	-	-	-	640	519	169.7	Arg389His/Arg389His		5(13)
12	F	+	-	-	-	343	263	147.4	Arg389His/Gly269Arg		6(13)
25	F	+	+	+	①	206	155	662.4	Glu22Ter/Glu22Ter		7(14)
24	F	+	+	+	-	220	151	1164.0	Glu22Ter/Glu22Ter		Sibling of case7(14)
23	M	+	+	+	-	135	89	861.6	Glu22Ter/Glu22Ter		Sibling of case7(14)
34	F	+	+	+	-	220	151	1380.0	Arg446Ter/Arg446Ter		10(14)
43	F	+	+	+	-	339	236	1879.2		Met614-Lys628del/Glu25Ter	11(14)
61	M	+	+	+	-	332	221	1173.6	g.9+2 A>G/Arg446Ter		12(14)
58	M	+	+	+	-	144	65	710.4	g.9+2 A>G /Arg446Ter		Sibling of case12(14)
57	M	+	+	+	-	348	224	564.0	g.9+2 A>G /Arg446Ter		Sibling of case12(14)
53	F	+	+	+	-	339	236	1485.6	g.9+2 A>G /Arg446Ter		Sibling of case12(14)
31	M	-	+	-	-	316	199	1653.6	Arg419His/Arg419His		16(14)
58	M	+	+	+	-	128	109	1608.0		Leu86Pro fs Ter185/ Leu86Pro fs Ter185	17(14)
45	F	+	+	+	-	228	109	2164.8	g.ISV7+3G>A/ g.ISV7+3G>A		18(14)
59	F	+	+	-	②	213	128	2092.8		Arg263Gln/Glu500Asp fs Ter604	19(14)
67	F	+	+	-	③⑨	183	NA	684.0	Gly90Glu/Arg389His		20(15)
60	M	+	-	-	-	221	NA	794.4		Gly674Arg/Gly674Arg	21(15)
49	F	+	+	-	④	203	NA	686.4		Gly674Arg /Gly674Arg	22(15)
45	F	-	-	-	-	191	NA	645.6		Gly674Arg/ Gly674Arg	23(15)
45	M	+	-	-	-	153	NA	422.4	g.7+2G>A/ g.7+2G>A		24(15)
44	F	+	-	-	④	213	NA	686.4	Arg446Ter / Arg446Ter		25(15)
39	F	+	-	-	-	NA	NA	NA	Arg389His/Arg389His		26(15)
15	F	+	-	-	-	291	178	582		Arg146Ter /Leu650Arg	27(16)
14	M	+	+	-	⑤⑥	412	332	516.9	Arg446Ter /Arg446Ter		28(17)
6.2	M	+	-	-	-	559	349	231.2		Arg263Gln/c.1528_1530delATC	29(18)
5	F	+	-	-	-	599	524	177.7	Asn437Lys/ Gln251Ter		30(18)
1.3	F	+	-	-	-	613	483	114.2	Arg446Ter / Gln251Ter		31(18)
9	M	+	-	-	-	557	430	NA	Arg389His/ Arg389His		32(19)
7	M	+	-	-	-	478	359	NA	Gln251Ter / Gln251Ter		33(19)
2	M	+	-	-	-	669	559	318.9	FM^b^		34(20)
2	F	+	-	-	-	942	735	132.5	FM		35(20)
2	F	+	-	-	-	448	401	469.7	FM		36(20)
5	M	+	-	-	-	755	569	392.7		FM	37(20)
7	M	+	-	-	①⑥	503	454	541.7	FM		38(20)
8	F	-	-	-	⑦	318	255	237.9	FM		Sibling of case35(20)
1	M	-	-	-	-	410	347	NA	FM	FM	40(20)
2	F	+	-	-	-	613	549	NA	FM		41(20)
2	M	+	-	-	-	593	483	NA		FM	42(20)
2	F	+	-	-	⑦	747	553	NA	FM		43(20)
3	F	+	-	-	①	565	472	NA		FM	44(20)
5	M	+	-	-	-	530	355	NA	FM		45(20)
5	M	-	-	-	-	389	240	NA		FM	46(20)
7	M	-	-	-	-	457	314	NA	FM		47(20)
7	M	+	-	-	-	549	369	NA		FM	48(20)
8	M	+	-	-	-	567	404	NA	FM		49(20)
9	F	+	-	-	⑧	772	606	NA	FM		50(20)
12	M	+	-	-	①⑥	468	338	355.8	FM		51(20)
1	F	+	-	+	-	800	549	20.6	Gln251Ter /Arg446Gln		52(21)
1.4	M	+	-	+	-	348	256	11.7	c.904+1G>A/c.-76 C>T		53(21)
8	M	-	+	+	-	239	146	32.2		c.965-1G>A/Ser473Ter	54(21)
3	F	+	-	-	-	483	414	9.9		c.323-1G>C/Gly512Arg	55(21)

①Carotid plaque; ②heart block; ③cardiac hypertrophy; ④premature beats; ⑤aortic valve stenosis; ⑥aortic valves regulation; ⑦mitral regurgitation; ⑧pulmonary stenosis; ⑨myocardial infarction

*Abbreviations:* TC total cholesterol, LDL-C low density lipoprotein cholesterol, M male, F female, NA not available, FM found mutation

^a^ +present, - absent

^b^ The article lists mutated genes but does not list specific sites

Among all the reported patients, 39 (71%) had *ABCG5* mutations, and 15 (27%) had *ABCG8* mutations. Only one patient (2%) had both *ABCG5* and *ABCG8* mutations. Some mutations have been reported previously, while several mutations were identified for the first time. Among these mutations, Arg446Ter, Gln251Ter, and Arg389His at *ABCG5* were present in 18 out of 27 patients (67%) with sitosterolemia (Table 2).

## Discussion

With complicated and varied clinical manifestations, it is difficult to diagnose sitoterolemia and treat in the early stage. Persistent high cholesterol levels can cause premature coronary artery disease; therefore, early diagnosis and treatment are very important for patients with sitosterolemia. For Chinese patients, the minimum age at which carotid artery plaques developed was 2 years old (patient 43). The correlation between blood levels of plant sterols and premature coronary artery disease is not clear. In patients with lipid disorders, lipoprotein (a) [Lp(a)] is an independent risk factor for cardiovascular disease [28]. High Lp(a) levels have been related to worse cardiovascular prognosis [29], However, Lp(a) was normal in the four patients described here and other reported patients [21].

Routine biochemical testing cannot distinguish phytosterols from cholesterol. The quantitative measurement of blood phytosterol concentrations, which requires special equipment and technology, is still the gold standard for the diagnosis of sitosterolemia. However, most local clinical laboratories may not have this equipment. In this study, the natural history and follow-up of four pediatric patients with sitosterolemia were described; and the clinical manifestations and gene mutations of 55 Chinese patients with sitosterolemia were analysed and summarized. One case (patient 2) of sitosterolemia combined with GSD VI was reported for the first time. The *ABCG5* and/or *ABCG8* genes were analyzed in all 55 patients in the previously studied Chinese cohort. There was no significant difference in the clinical symptoms between the Chinese and Caucasian patients. In contrast to mainly variations in *ABCG8* gene in Caucasians, most Chinese patients have mutations in *ABCG5* [30, 31], and Arg446Ter, Gln251Ter, and Arg389His might be hot-spot mutations in Chinese patients.

The majority of patients with sitosterolemia had delayed diagnosis, the delay from onset to diagnosis had been reported to be up to 28.8 years [14, 15]. In the present cohort, the mean age of symptom onset was 2.2 years, and the mean age of diagnosis was 6.4 years. Misdiagnosis often led to inappropriate treatment. Twenty Chinese patients with splenomegaly underwent a splenectomy at the ages of 9 to 61 years old [15]. The symptoms of anemia were greatly improved after splenectomy in patients with sitosterolemia, but platelet counts remained persistently low. Some patients with thrombocytopenia were administered steroids [14, 30]. Both patient 1 and patient 3 were misdiagnosed with FH and were treated with statins, resulting in worsening of the symptoms after 1 to 2 years of therapy.

Hybrid heterozygous mutations were identified in both the *PYGL* and *ABCG5* genes by WES in patient 2, and Sanger sequencing verification was performed. The missense mutation p.Glu383Asp of *PYGL*, occurring in a highly conserved residue, was a novel mutation and was predicted to be damaging using the SIFT algorithm, Polyphen-2, and Mutation-Assessor for protein functions. Replacement of the amino acids might affect the stability of the protein structure, which might disrupt the positioning and binding of protein substrates and their catalytic sites. The other detected PYGL mutation, gross deletion of PYGL exons 14–17, was previously reported in a Chinese patient [32], *PYGL* exons 14 to 17 encode approximately one-fifth of the length of the protein.

Xanthomas were observed in only 2 out of the 4 patients (patient 1 and patient 4). Only patient 2 had hepatomegaly, significant liver enzyme elevation, high TGs, mild elevation of lactic acid and ketosis which are consistent with the diagnosis of GSD VI, in addition to high TC. She was treated with uncooked cornstarch and responded very well to the treatment as her liver size, liver transaminases, triglycerides and lactic acid were all normalized and remained normal on follow-up at 3 years after treatment was started. No hypoglycemia was detected during the entire course of the disease. Partial catch-up growth was observed with her height and weight crossing up centile lines from 3rd to 10th percentiles before treatment to the 25th percentile 3 years after the treatment was started. However, the plant sterols remained elevated. The catch-up growth she demonstrated with the treatment suggested that GSD VI, rather than sitosterolemia, was the main factor affecting her growth and development.

Regarding the drug treatment of sitosterolemia, some Chinese patients were treated with ezetimibe at a dose of 5 mg/d or 10 mg/d. The majority showed good response to this therapy. However, the infant who received ezetimibe at an early age of 3 months did not respond to the treatment. Another very young patient who was started on ezetimibe treatment at the age of 2 years showed a slower response than the adult patients with sitosterolemia [13–15, 33].

In this cohort, patients 1 and 3 received dietary restriction combined with cholestyramine, while patients 2 and 4 underwent dietary treatment alone. Eight months to 3 years of follow-up of the patients revealed that TC levels remained in the normal range and xanthomas disappeared gradually after combined dietary and cholestyramine treatment. Nevertheless, the levels of plant sterols remained high. Dietary treatment alone failed in the two patients, especially for the younger patient 4 (Table 1). Different plant sterol intakes due to different diets might be the main cause of the unsatisfactory effect of treatment in infantile patients. However, it cannot be concluded whether the poor response to dietary treatment in patient 2 is related to GSD VI. Uncooked cornstarch contains a high amount of plant sterol, up to 60 mg/100 g [34], while sitosterolemia requires a low-phytosterol diet. Hence, it is a great challenge to balance them.

**Fig. 2:**
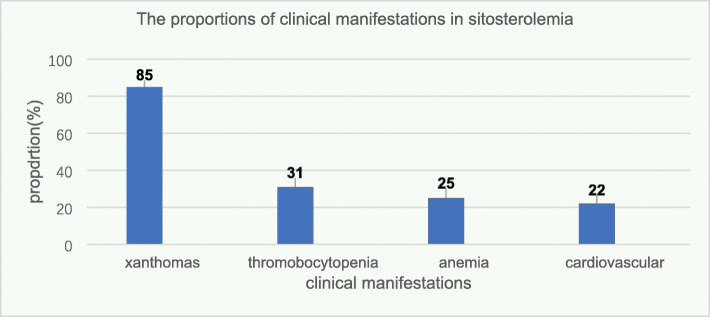
The sitosterolemia diagnosis and treatment flowchart

In conclusion, although pharmacotherapy including ezetimibe and cholestyramine has been established as the standard of care, there is still no consensus on sitosterolemia treatment, especially for children under 2 years old. Treatment should be started immediately at diagnosis. Limiting phytosterols and cholesterol should be the first strategy (Fig. 2) [35, 36]. For patients with an incomplete response to ezetimibe, combination with cholestyramine may be a feasible additional treatment [11]. The treatment experience in this cohort suggested that a significant effect cannot be achieved with a small dose of cholestyramine. However, more studies are required to examine whether to combine the drugs or to increase the dose. Based on the current therapeutic outcomes, after obtaining parental consent, ezetimibe was started in the four patients. An ongoing study is expected to provide further information.

Misdiagnosis still frequently occurs for sitosterolemia in China, and many patients undergo unnecessary operations due to misdiagnosis. However, there is no unified diagnostic criteria for sitosterolemia. Measurement of plasma plant sterols should be considered in patients with considerably elevated cholesterol levels and negative genetic tests for FH, especially when there is concomitant xanthomatosis.

### Comparisons with other studies and what does the current work add to the existing knowledge

Most previous studies [12, 14–18, 21] were case reports, and most of the Chinese patients did not have data on treatment and follow-up. Currently, there is no study summarizing the clinical characteristics and gene mutation types of Chinese sitosterolemia patients. In this study, an in-depth summary of the phenotype and genotype of sitosterolemia patients in China is provided. Notably, four pediatric sitosterolemia cases are described, and in particular, one case featuring a concomitant GSD VI mutation is reported for the first time.

## Study strengths and limitations

Currently, sitosterolemia pediatric cases in China have rarely been reported. The strength of the current study is that it comprehensively collected related information for sitosterolemia patients among Chinese patients and summarized the data in depth to provide meaningful guidance for the treatment of such diseases in the clinic. The limitations of this study are small sample size and the short follow-up visit periods, in particularly for patients 2 and 4. To address the shortcomings, further follow-up visits and systematic monitoring for all four pediatric patients will be conducted.

## Conclusions

This study reported four patients with sitosterolemia and reviewed a total of 55 Chinese patients. Morbidity can occur early in life for most patients. Therefore, it is essential to conduct plasma phytosterol assays for patients featuring early onset xanthomas and hypercholesterolemia with poor response to statins. Special attention should be given to patients with previous morbidity of coronary artery disease as well as patients presenting with hematological symptoms of macrothrombocytopenia and stomatocytic hemolysis. Early diagnosis and treatment can prevent complications and improve the prognosis for patients.

## Data Availability

The datasets used and/or analyzed during the current study are available from the corresponding author on reasonable request.

## References

[1] Patel SB, Salen G, Hidaka H, Kwiterovich PO, Stalenhoef AF (1998). Mapping a gene involved in regulating dietary cholesterol absorption. The sitosterolemia locus is found at chromosome 2p21. J Clin Invest.

[2] Berge KE, Tian H, Graf GA, Yu L, Grishin NV, Schultz J (2000). Accumulation of dietary cholesterol in sitosterolemia caused by mutations in adjacent ABC transporters. Science.

[3] Lee MH, Lu K, Hazard S, Yu H, Shulenin S, Hidaka H (2001). Identification of a gene, ABCG5, important in the regulation of dietary cholesterol absorption. Nat Genet.

[4] Rees DC, Iolascon A, Carella M, O’marcaigh AS, Kendra JR (2005). Stomatocytic hemolysis and macrothrombocytopenia (Mediterranean stomatocytosis/macrothrombocytopenia) is the hematological presentation of phytosterolaemia. Br J Haematol.

[5] Wang Z, Cao L, Su Y (2014). Specific macrothrombocytopenia/hemolytic anemia associated with sitosterolemia. Am J Hematol.

[6] Yoo EG (2016). Sitosterolemia: a review and update of pathophysiology, clinical spectrum, diagnosis, and management. Ann Pediatr Endocrinol Metab.

[7] Escolà-Gil JC, Quesada H, Julve J, Martín-Campos JM, Cedó L, Blanco-Vaca F (2014). Sitosterolemia: diagnosis, investigation, and management. Curr Atheroscler Rep.

[8] Othman RA, Myrie SB, Jones PJ (2013). Non-cholesterol sterols and cholesterol metabolism in sitosterolemia. Atherosclerosis.

[9] Tsubakio-Yamamoto K, Nishida M, Nakagawa-Toyama Y, Masuda D, Ohama T, Yamashita S (2010). Current therapy for patients with sitosterolemia – effect of ezetimibe on plant sterol metabolism. J Atheroscler Thromb.

[10] Sudhop T, Lütjohann D, von Bergmann K (2005). Sterol transporters: targets of natural sterols and new lipid lowering drugs. Pharmacol Ther.

[11] Salen G, Starc T, Sisk CM, Patel SB (2006). Intestinal cholesterol absorption inhibitor ezetimibe added to cholestyramine for sitosterolemia and xanthomatosis. Gastroenterology.

[12] Lam CW, Cheng AW, Tong SF, Chan YW (2002). Novel donor splice site mutation of ABCG5 gene in sitosterolemia. Mol Genet Metab.

[13] Niu DM, Chong KW, Hsu JH, Wu TJ, Yu HC, Huang CH, Lo MY, Kwok CF, Kratz LE, Ho LT (2010). Clinical observations, molecular genetic analysis, and treatment of sitosterolemia in infants and children. J Inherit Metab Dis.

[14] Wang Z, Cao L, Su Y, Wang G, Wang R, Yu Z, Bai X, Ruan C (2014). Specific macrothrombocytopenia/hemolytic anemia associated with sitosterolemia. Am J Hematol.

[15] Cao LJ, Yu ZJ, Jiang M, Bai X, Su J, Dai L, Ruan CG, Wang ZY (2019). Clinical features of 20 patients with phytosterolemia causing hematologic abnormalities. Zhonghua Yi Xue Za Zhi..

[16] Hu M, Yuen YP, Kwok JS, Griffith JF, Tomlinson B (2014). Potential effects of NPC1L1 polymorphisms in protecting against clinical disease in a chinese family with sitosterolaemia. J Atheroscler Thromb.

[17] Wang Y, Guo YL, Dong QT, Li JJ (2019). Severe aortic valve stenosis in a 14-year-old boy with sitosterolemia. J Clin Lipidol.

[18] Fang D, Liang LL, Qiu WJ, Fan YJ, Sun Y, Yan H, Yu YG, Gu XF (2018). Clinical, molecular genetic analysis, and treatment of 3 children with sitosterolemia. Zhonghua Er Ke Za Zhi..

[19] Huang D, Zhou Q, Chao YQ, Zou CC (2019). Clinical features and genetic analysis of childhood sitosterolemia: Two case reports and literature review. Medicine.

[20] Xu L, Wen W, Yang Y, Xie J, Li R, Wu Y, Hu Y, Wang L, Chong M (2020). Features of Sitosterolemia in Children. Am J Cardiol..

[21] Sun W, Zhang T, Zhang X, Wang J, Chen Y, Long Y (2020). Compound heterozygous mutations in ABCG5 or ABCG8 causing Chinese familial Sitosterolemia. J Gene Med.

[22] Beauchamp NJ, Taybert J, Champion MP, Layet V, Heinz-Erian P (2007). High frequency of missense mutations in glycogen storage disease type VI. J Inherit Metab Dis.

[23] Kishnani PS, Goldstein J, Austin SL, Arn P, Bachrach B (2019). ACMG Work Group on Diagnosis and Management of Glycogen Storage Diseases Type VI and IX. Diagnosis and management of glycogen storage diseases type VI and IX: a clinical practice resource of the American College of Medical Genetics and Genomics (ACMG). Genet Med.

[24] Nakai A, Shigematsu Y, Takano T, Kikawa Y, Sudo M (1994). Uncooked cornstarch treatment for hepatic phosphorylase kinase deficiency. Eur J Pediatr.

[25] Bratinčević MV, Visković T, Sutlović D (2017). Comparison of the solid phase and liquid-liquid extraction methods for methadone determination in human serum and whole blood samples using gas chromatography/mass spectrometry. Arh Hig Rada Toksikol..

[26] Hubacek JA, Berge KE, Cohen JC, Hobbs HH (2001). Mutations in ATP-cassette binding proteins G5 (ABCG5) and G8 (ABCG8) causing sitosterolemia. Hum Mutat.

[27] Escolà-Gil JC, Quesada H, Julve J, Martín-Campos JM, Cedó L, Blanco-Vaca F (2014). Sitosterolemia: diagnosis, investigation, and management. Curr Atheroscler Rep.

[28] Cesaro A, Schiavo A, Moscarella E, Coletta S, Conte M, Gragnano F (2021). Lipoprotein(a): a genetic marker for cardiovascular disease and target for emerging therapies. J Cardiovasc Med (Hagerstown)..

[29] Gragnano F, Fimiani F, Di Maio M, Cesaro A, Limongelli G, Cattano D, Calabrò P (2019). Impact of lipoprotein(a) levels on recurrent cardiovascular events in patients with premature coronary artery disease. Intern Emerg Med.

[30] Berge KE, Tian H, Graf GA, Yu L, Grishin NV (2000). Accumulation of dietary cholesterol in sitosterolemia caused by mutations in adjacent ABC transporters. Science..

[31] Niu DM, Chong KW, Hsu JH, Wu TJ, Yu HC (2010). Clinical observations, molecular genetic analysis, and treatment of sitosterolemia in infants and children. J Inherit Metab Dis.

[32] Luo X, Hu J, Gao X, Fan Y, Sun Y, Gu X, Qiu W (2020). Novel PYGL mutations in Chinese children leading to glycogen storage disease type VI: two case reports. BMC Med Genet..

[33] Salen G, von Bergmann K, Lütjohann D, Kwiterovich P, Kane J, Patel SB, Musliner T, Stein P, Musser B, Multicenter Sitosterolemia Study Group. Ezetimibe effectively reduces plasma plant sterols in patients with sitosterolemia. Circulation. 2004;109(8):71-966. 10.1161/01.CIR.0000116766.31036.03 PMC1237008 14769702

[34] Han J, Yang Y, Feng M, Wang G (2007). [Analysis of phytosterol contents in Chinese plant food and primar estimation of its intake of people]. Wei Sheng Yan Jiu.

[35] Bastida JM, Girós ML, Benito R, Janusz K, Hernández-Rivas JM, González-Porras JR (2019). Sitosterolemia: Diagnosis, Metabolic and Hematological Abnormalities, Cardiovascular Disease and Management. Curr Med Chem.

[36] Tada H, Nomura A, Ogura M, Ikewaki K, Ishigaki Y (2021). Diagnosis and Management of Sitosterolemia 2021. J Atheroscler Thromb..

